# Supplementary material for: Identification of a characteristic vascular belt zone in human colorectal cancer
Source: PLoS One. 2017 Mar 2;12(3):e0171378. doi: 10.1371/journal.pone.0171378 (PMC5333981; doi:10.1371/journal.pone.0171378)
Supplement: S1 Table — Sample IDs starting with “C2” are from the second cohort (validation cohort). Four samples from the primary cohort were excluded after staining (these are not shown): Smp042 was an appendix carcinoma, Smp055 was a lymph node metastasis, Smp059 was a duplicate of Smp058 and Smp064 was insufficiently stained and were removed after assignment of sample numbers. T = local tumor stage, N = lymph node stage, M = distant metastasis stage at time of surgery. In total, N = 100 samples were analyzed. (DOCX) [file pone.0171378.s001.docx]

| ID | | type | T | N | M |
| --- | --- | --- | --- | --- | --- |
| Smp001 | | met | - | - | - |
| Smp002 | | neo | ypT2 | ypN0 | yMX |
| Smp003 | | met | - | - | - |
| Smp004 | | met | - | - | - |
| Smp005 | | met | - | - | - |
| Smp006 | | met | - | - | - |
| Smp007 | | met | - | - | - |
| Smp008 | | neo | ypT3 | ypN2 | yM1 |
| Smp009 | | prim | pT3 | pN1 | M1 |
| Smp010 | | prim | pT2 | pN0 | M0 |
| Smp011 | | prim | pT3 | pN0 | MX |
| Smp012 | | prim | pT3 | pN0 | M0 |
| Smp013 | | prim | pT2 | pN0 | MX |
| Smp014 | | prim | pT3 | pN2 | M0 |
| Smp015 | | prim | pT4 | pN2 | M1 |
| Smp016 | | prim | pT4 | pN0 | MX |
| Smp017 | | prim | pT4 | pN1 | M1 |
| Smp018 | | prim | pT3 | pN0 | MX |
| Smp019 | | prim | pT3 | pN0 | M1 |
| Smp020 | | prim | pT3 | pN1 | MX |
| Smp021 | | prim | pT2 | pN1 | MX |
| Smp022 | | prim | pT3 | pN2 | M0 |
| Smp023 | | prim | pT3 | pN1 | M0 |
| Smp024 | | prim | pT2 | pN0 | M0 |
| Smp025 | | prim | pT2 | pN0 | M0 |
| Smp026 | | prim | pT3 | pN1 | M0 |
| Smp027 | | prim | pT4 | pN1 | M1 |
| Smp028 | | neo | ypT2 | ypN0 | yM0 |
| Smp029 | | prim | pT3 | pN0 | M1 |
| Smp030 | | prim | pT3 | pN0 | M0 |
| Smp031 | | prim | pT3 | pN0 | M0 |
| Smp032 | | prim | pT2 | pN0 | M0 |
| Smp033 | | prim | pT3 | pN2 | M0 |
| Smp034 | | prim | pT2 | pN0 | MX |
| Smp035 | | prim | pT3 | pN0 | M1 |
| Smp036 | | prim | pT2 | pN0 | MX |
| Smp037 | | met | - | - | - |
| Smp038 | | met | - | - | - |
| Smp039 | | prim | pT3 | pN1 | M1 |
| Smp040 | | prim | pT4 | pN2 | MX |
| Smp041 | | prim | pT4 | pN2 | M1 |
| Smp043 | | prim | pT4 | pN2 | M1 |
| Smp044 | | met | - | - | - |
| Smp045 | | prim | pT4 | pN1 | MX |
| Smp046 | | prim | pT3 | pNX | MX |
| Smp047 | | prim | pT4 | pN0 | M1 |
| Smp048 | | neo | ypT3 | ypN1 | yM1 |
| Smp049 | | prim | pT4 | pN2 | MX |
| Smp050 | | prim | pT4 | pN2 | M1 |
| Smp051 | | prim | pT3 | pN0 | MX |
| Smp052 | | prim | pT3 | pN1 | M0 |
| Smp053 | | prim | pT3 | pN2 | M0 |
| Smp054 | | prim | pT2 | pN1 | M0 |
| Smp056 | | prim | pT3 | pN1 | M0 |
| Smp057 | | prim | pT2 | pN0 | M0 |
| Smp058 | | prim | pT2 | pN1 | M0 |
| Smp060 | | neo | ypT2 | ypN0 | yM0 |
| Smp061 | | prim | pT3 | pN1 | MX |
| Smp062 | | prim | pT1 | pN0 | M0 |
| Smp063 | | met | - | - | - |
| Smp065 | | met | - | - | - |
| C2-Smp001 | | prim | pT2 | pN0 | MX |
| C2-Smp002 | | prim | pT3 | pN0 | MX |
| C2-Smp003 | | prim | pT3 | pN1 | MX |
| C2-Smp004 | | prim | pT3 | pN0 | MX |
| C2-Smp005 | | prim | pT2 | pN1 | MX |
| C2-Smp006 | | prim | pT3 | pN2 | M1 |
| C2-Smp007 | | prim | pT3 | pN1 | MX |
| C2-Smp008 | | prim | pT2 | pN0 | MX |
| C2-Smp009 | | prim | pT4 | pN1 | M1 |
| C2-Smp010 | | prim | pT3 | pN0 | MX |
| C2-Smp011 | | prim | pT2 | pN0 | MX |
| C2-Smp012 | | prim | pT3 | pN0 | MX |
| C2-Smp013 | | prim | pT2 | pN0 | MX |
| C2-Smp014 | | prim | pT3 | pN1 | M1 |
| C2-Smp015 | | prim | pT2 | pN0 | MX |
| C2-Smp016 | | prim | pT3 | pN1 | MX |
| C2-Smp017 | | prim | pT1 | pN0 | MX |
| C2-Smp018 | | prim | pT4 | pN0 | MX |
| C2-Smp019 | | prim | pT3 | pN0 | MX |
| C2-Smp020 | | prim | pT2 | pN0 | MX |
| C2-Smp021 | | prim | pT3 | pN2 | MX |
| C2-Smp022 | | prim | pT3 | pN0 | MX |
| C2-Smp023 | | prim | pT2 | pN0 | MX |
| C2-Smp024 | met | | - | - | - |
| C2-Smp025 | met | | - | - | - |
| C2-Smp026 | met | | - | - | - |
| C2-Smp027 | met | | - | - | - |
| C2-Smp028 | met | | - | - | - |
| C2-Smp029 | met | | - | - | - |
| C2-Smp030 | met | | - | - | - |
| C2-Smp031 | met | | - | - | - |
| C2-Smp032 | met | | - | - | - |
| C2-Smp033 | met | | - | - | - |
| C2-Smp034 | met | | - | - | - |
| C2-Smp035 | met | | - | - | - |
| C2-Smp036 | met | | - | - | - |
| C2-Smp037 | met | | - | - | - |
| C2-Smp038 | met | | - | - | - |
| C2-Smp039 | met | | - | - | - |
