# Supplementary figures and images for: Identification of a characteristic vascular belt zone in human colorectal cancer
Source: PLoS One. 2017 Mar 2;12(3):e0171378. doi: 10.1371/journal.pone.0171378 (PMC5333981; doi:10.1371/journal.pone.0171378)

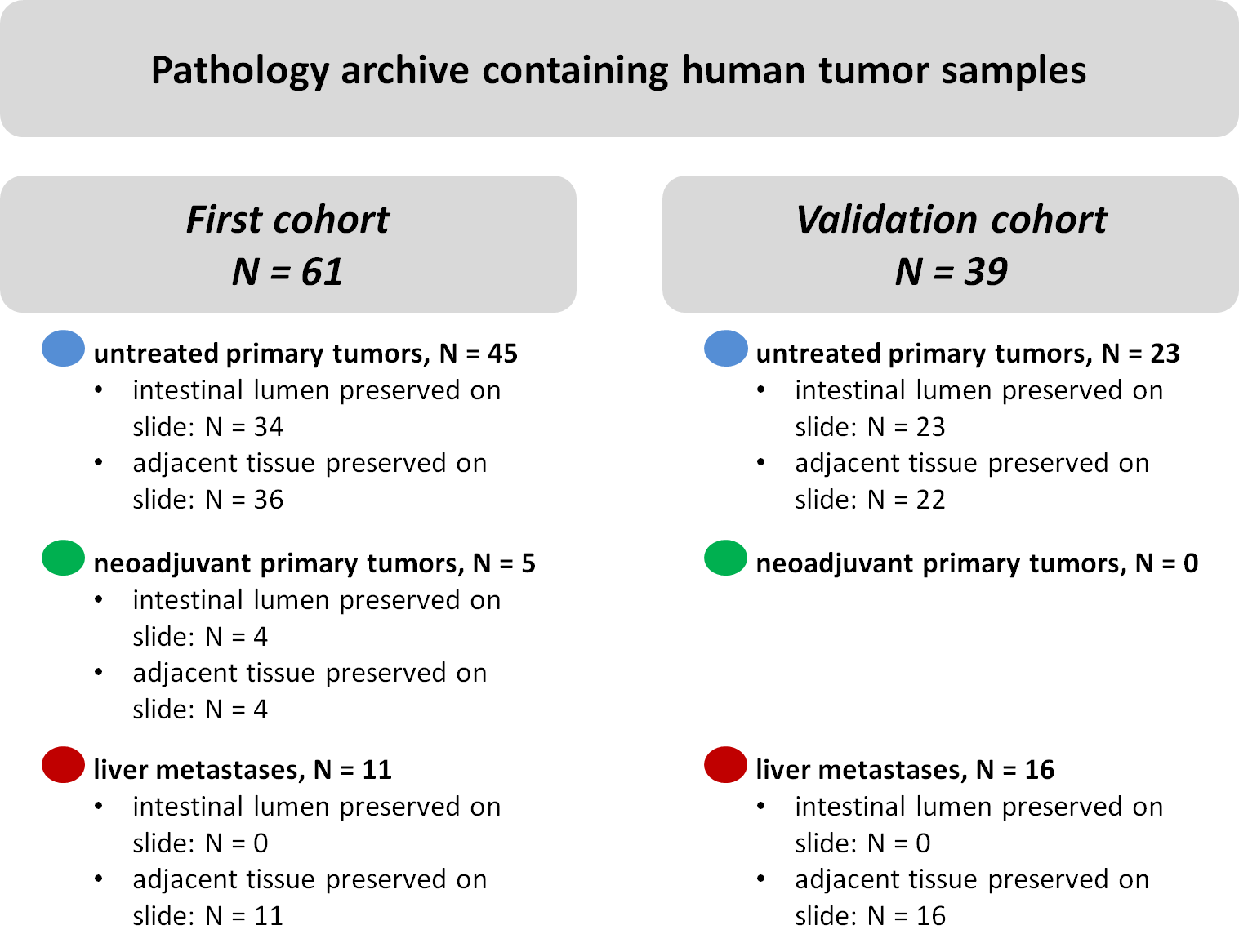

Supplement: S1 Fig — Here, the two cohorts are schematically shown. (TIF) [file pone.0171378.s002.tif]

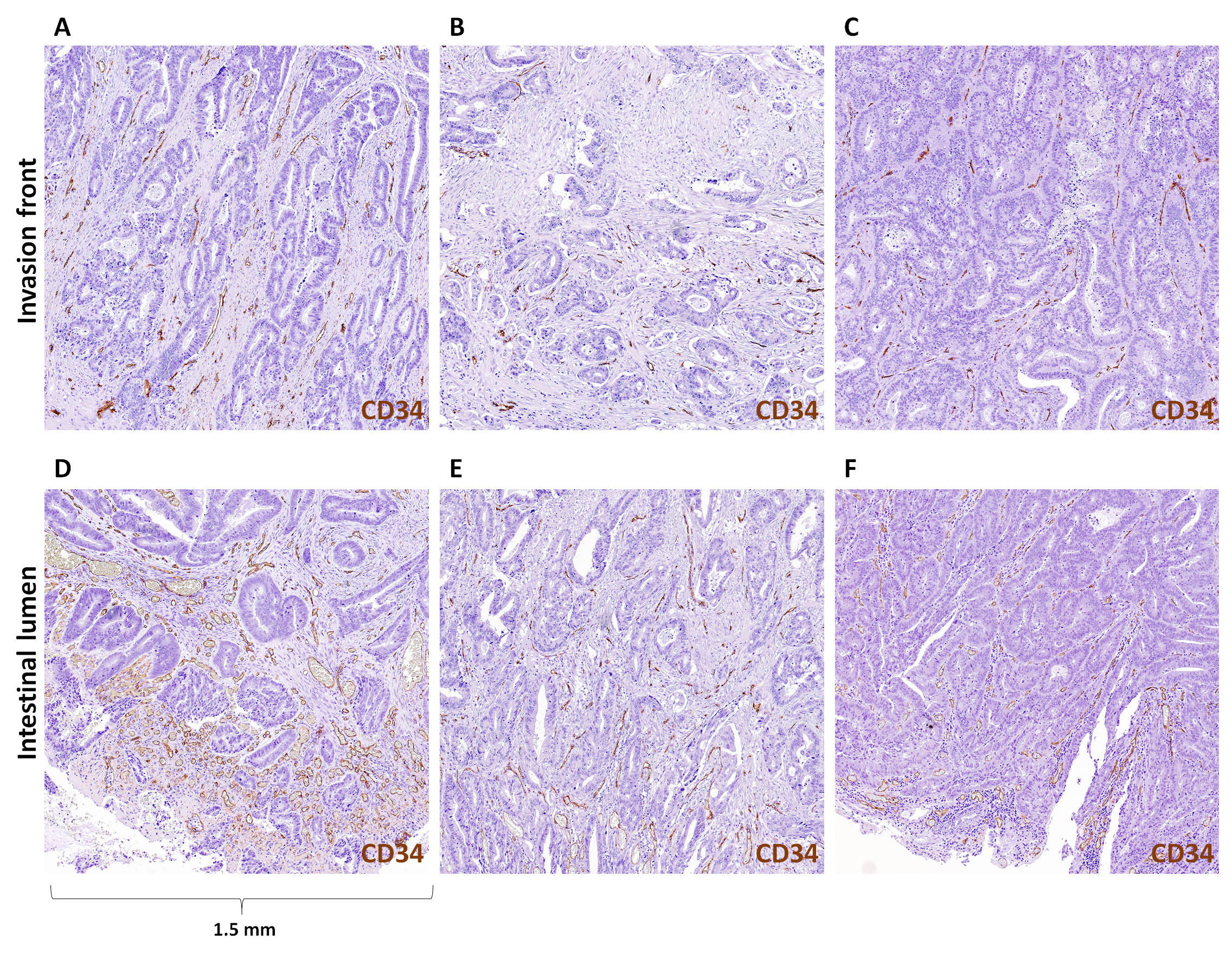

Supplement: S2 Fig — Image patches of 2.25 mm2 close to the intestinal lumen and close to the invasion front are shown, with A matching D, B matching E and C matching F. All three samples are CRC primary tumors. Even in this low magnification, blood vessels (CD34-positive) are much more abundant in the image patches close to the intestinal lumen. As seen in D (and, to a lesser extent, in E and F), these blood vessels are usually large and dilated. (TIF) [file pone.0171378.s003.tif]

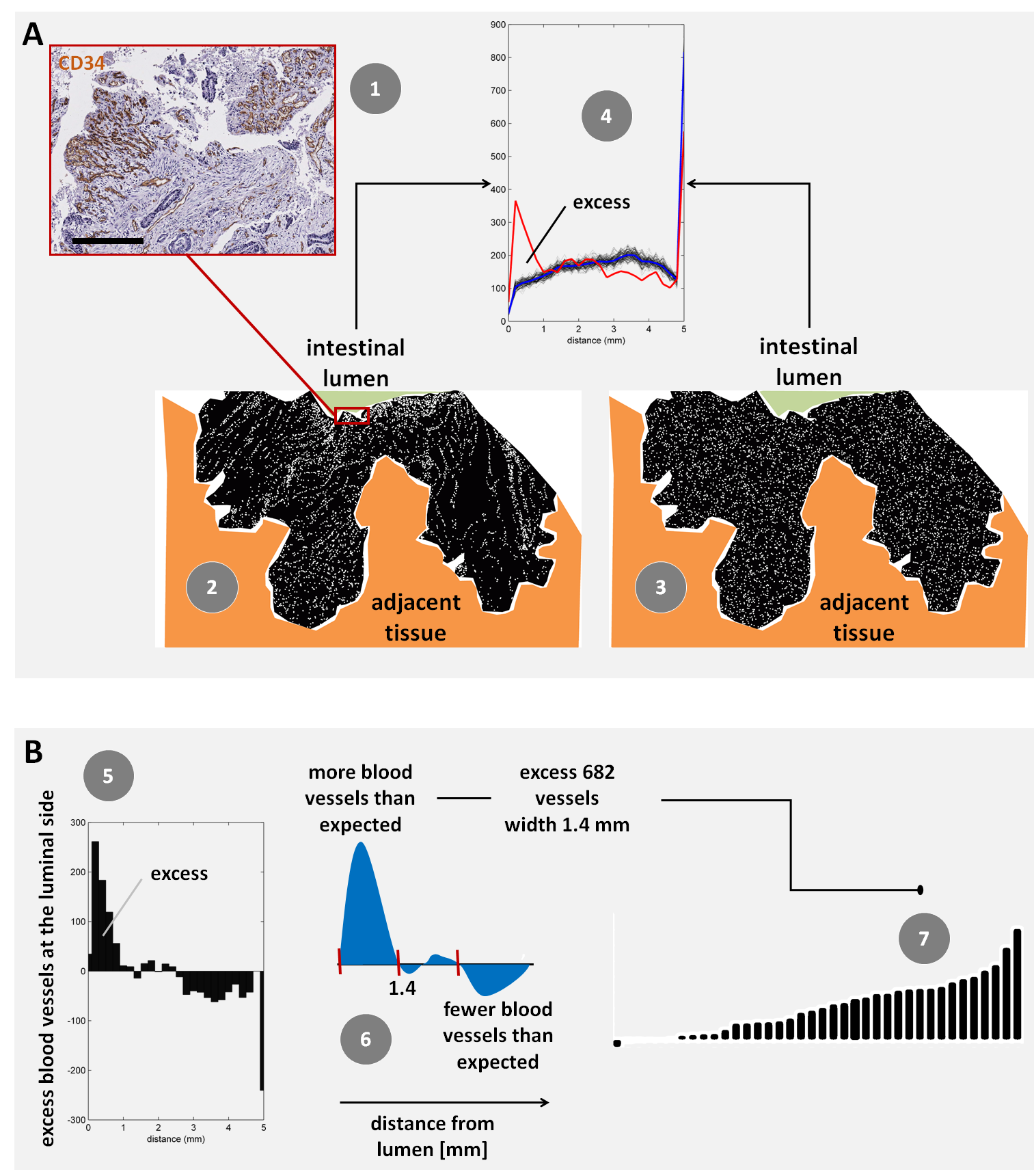

Supplement: S3 Fig — (A) Comparison of observed spatial distribution patterns to random patterns. (1) Detail from the original WSI (Smp040), scale bar 300 μm, CD34-immunostaining labels blood vessel endothelium. The area is densely packed with blood vessels. (2) In the WSI, three regions are manually defined: intestinal lumen (green), tumor (black), adjacent tissue (orange). Blood vessels in the WSI are automatically detected and plotted as white points. (3) To generate an internal control, detected blood vessels in a ROI are randomly redistributed N = 100 times using a Monte Carlo procedure so that overall MVD remains constant. (4) For each blood vessel, the distance to the intestinal lumen is calculated. This is repeated for each random pattern. The actual distribution of distances is compared to the distribution of random distances, plotted as a histogram. (B) Identification of excess zones: (5) The distance distribution of the random patterns is subtracted from the distance distribution of the observed patterns. The resulting difference is plotted as an excess histogram E(x). The first peak in E(x) reflects an excess of blood vessels at the luminal side. (6) The width of this peak describes the width of the excess zone while the total peak area describes the excess amount. (7) The excess amount is represented as one bar in a waterfall plot. (TIF) [file pone.0171378.s004.tif]

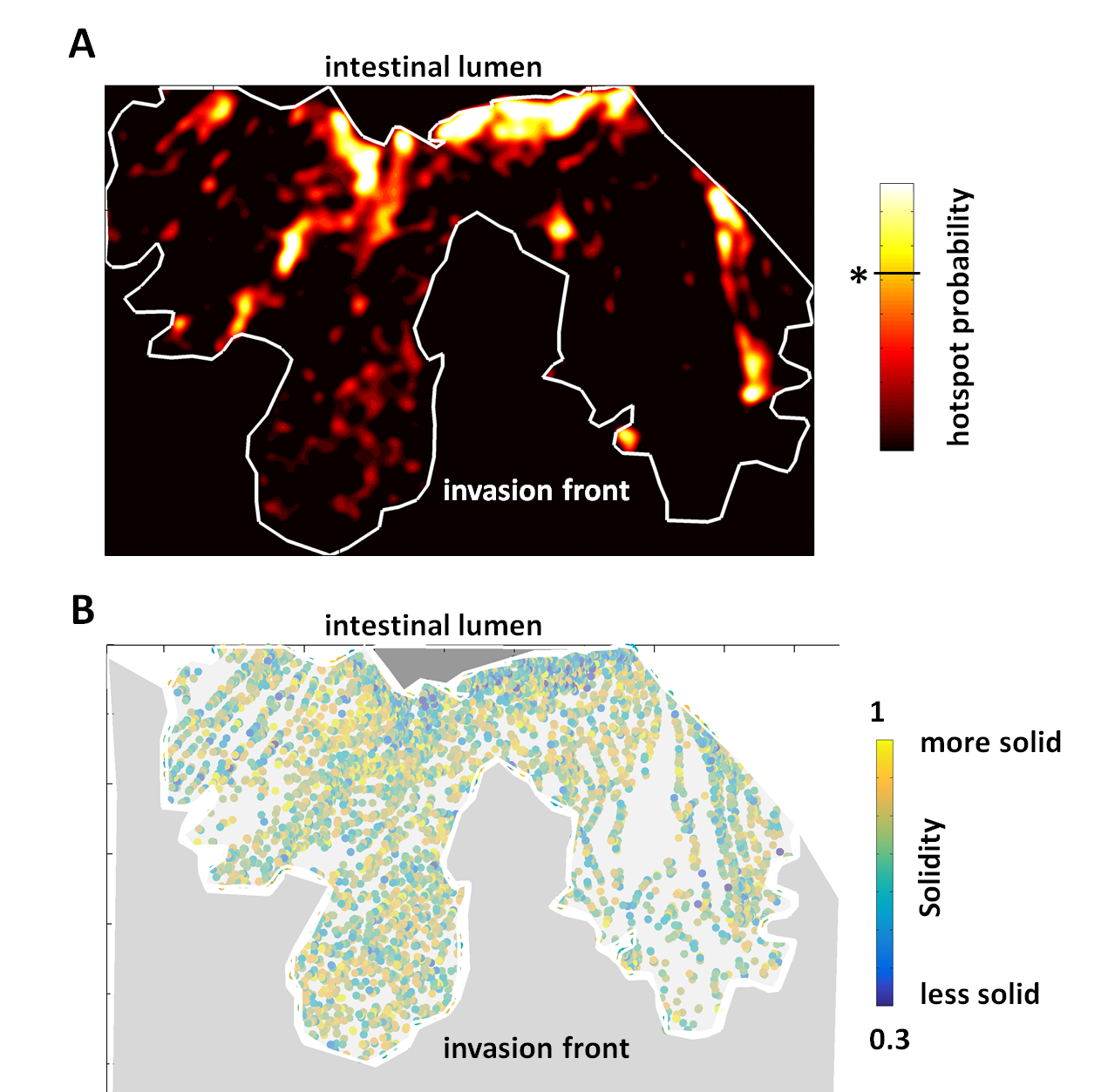

Supplement: S4 Fig — (A) Angiogenic hotspot probability map in a representative sample (Smp040), (*) refers to the level of significance. (B) Each blood vessel within the tumor is represented by a filled circle. The bright gray region shows the adjacent tissue and the dark gray region shows the intestinal lumen. As shown above, blood vessels tend to cluster at the intestinal lumen. Additionally, the solidity (defined as area divided by convex area) is shown for each blood vessel (blue = less solid, yellow = solid). Most non-solid vessels are located at the luminal side. This corresponds to the morphological observation that intratumoral blood vessels at the intestinal lumen tend to be more dilated than blood vessels in other tumor regions. (TIF) [file pone.0171378.s005.tif]

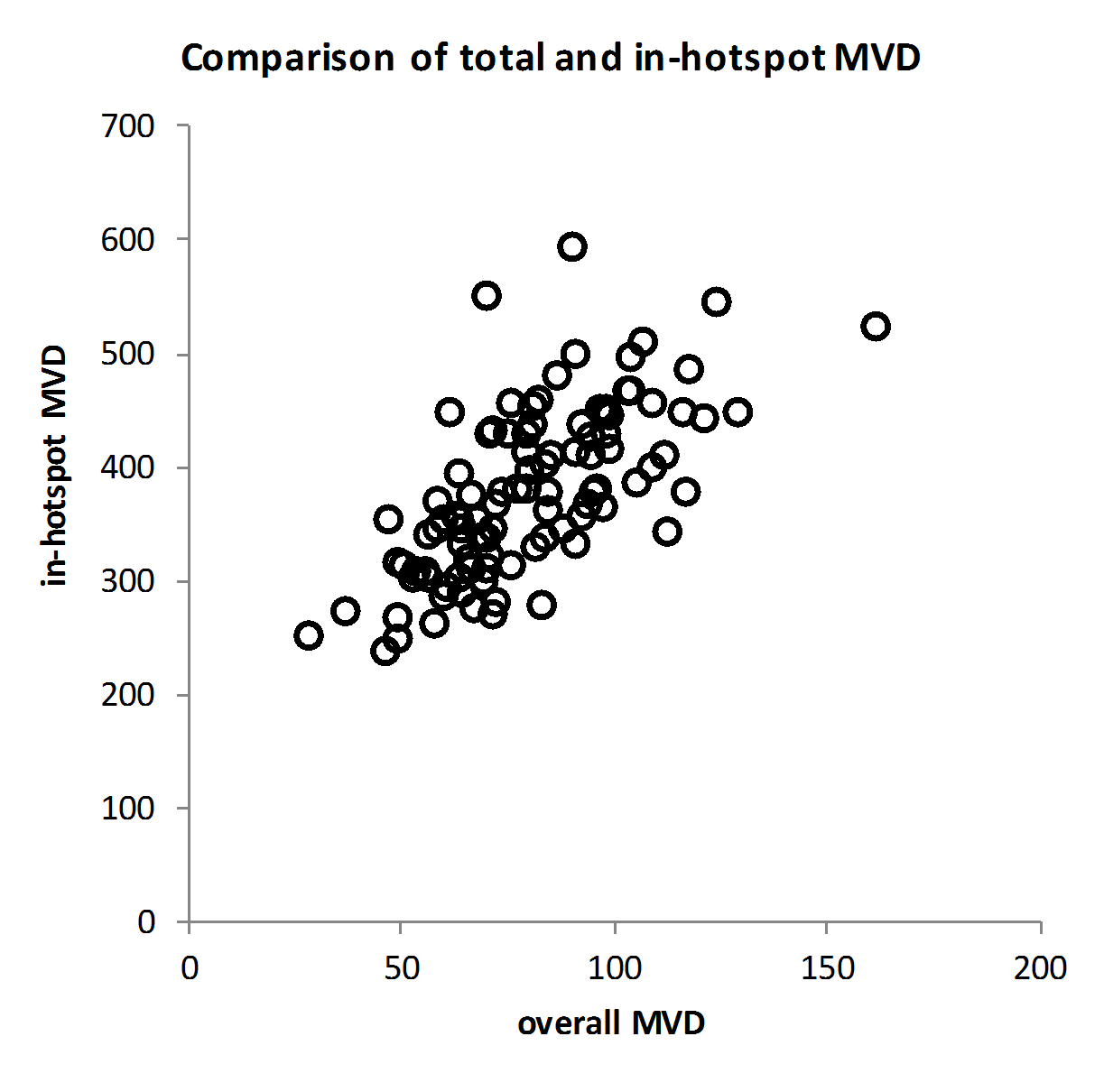

Supplement: S5 Fig — Scatter plot of overall MVD vs. in-hotspot of all samples (N = 100, both cohorts). Pearson’s correlation coefficient is r = 0.67. (TIF) [file pone.0171378.s006.tif]
