# Supplementary material for: Identification of a characteristic vascular belt zone in human colorectal cancer
Source: PLoS One. 2017 Mar 2;12(3):e0171378. doi: 10.1371/journal.pone.0171378 (PMC5333981; doi:10.1371/journal.pone.0171378)
Supplement: S1 List — (DOCX) [file pone.0171378.s007.docx]

- Resize factor for images prior to object recognition: 0.2
- TIFF block size: 240 px, TIFF scale 0.50 µm/pxl (range of 0.4948 and 0.5041 µm/px)
- Image block size for parallel processing of CD34-stained WSI: 5000 x 5000 px, overlap 300 x 300 px
- Smoothing kernel for binary object mask: dilation 9 x 9 px, erosion 5 x 5 px
- Fixed KDE kernel bandwidth for density functions: 190 px = 94 µm (as calculated in [[1](#_ENREF_1)], adjusted to image scaling in the present study)
- Seed for random number generator before each experimental set: 3
- Repetitions of the random experiments: 100
- Max. distance assessed in the distance histogram: 5 mm. To identify the extent of a positive or negative peak in the histogram E(x), we identified the first x-intersection (disregarding the first bin of 200 µm width).
- Delineation of “Tumor” / “Lumen” / “Adjacent Tissue” ROIs: The border was drawn tightly to the tumor epithelium; soft-tissue between tumor formations was regarded as tumor stroma and soft tissue adjacent to the tumor formations but not between them was regarded as peritumoral stroma / peritumoral fibrotic reaction.
