# Supplementary material for: Correction: Identification of a characteristic vascular belt zone in human colorectal cancer
Source: PLoS One. 2017 May 8;12(5):e0177586. doi: 10.1371/journal.pone.0177586 (PMC5421812; doi:10.1371/journal.pone.0177586)
Supplement: S2 File — (PDF) [file pone.0177586.s002.pdf]

PLOS | ONE

RESEARCH ARTICLE

Identification of a characteristic vascular belt zone in human colorectal cancer

Jakob Nikolas Kather[1,2,3]*, Frank Gerrit Zöllner[3], Lothar R. Schad[3], Susanne Maria Melchers[4], Hans-Peter Sinn[5], Alexander Marx[2], Timo Gaiser[2], Cleo-Aron Weis[2]

1 Department of Medical Oncology and Internal Medicine VI, National Center for Tumor Diseases, University Hospital Heidelberg, Heidelberg University, Heidelberg, Germany, **2** Institute of Pathology, University Medical Center Mannheim, Heidelberg University, Mannheim, Germany, **3** Institute of Computer Assisted Clinical Medicine, Medical Faculty Mannheim, Heidelberg University, Mannheim, Germany, **4** Department of Dermatology, Venereology and Allergology, University Medical Center Mannheim, Heidelberg University, Mannheim, Germany, **5** Department of Pathology, University Hospital Heidelberg, Heidelberg University, Heidelberg, Germany

* jakob.kather@nct-heidelberg.de

Check for updates

OPEN ACCESS

Citation: Kather JN, Zöllner FG, Schad LR, Melchers SM, Sinn H-P, Marx A, et al. (2017) Identification of a characteristic vascular belt zone in human colorectal cancer. PLoS ONE 12(3): e0171378. doi:10.1371/journal.pone.0171378

Editor: Domenico Ribatti, Universita degli Studi di Bari Aldo Moro, ITALY

Received: September 8, 2016

Accepted: January 18, 2017

Published: March 2, 2017

Copyright: © 2017 Kather et al. This is an open access article distributed under the terms of the Creative Commons Attribution License, which permits unrestricted use, distribution, and reproduction in any medium, provided the original author and source are credited.

Data Availability Statement: The data can be accessed via the following DOI: 10.17605/OSF.IO/D8AR6. This includes raw TIFF images of all N=100 CRC samples and TIFF images of N=5 normal colon mucosa. Furthermore, we release N=100 histological images that were used for validation of the segmentation procedure by human observers: DOI: 10.5281/zenodo.117997. This dataset includes the counts of three blinded observers and can be used by other groups to validate their own blood vessel segmentation algorithms. Source codes used for this study are available under the MIT license (http://opensource.org/licenses/MIT)

Abstract

Blood vessels in cancer

Intra-tumoral blood vessels are of supreme importance for tumor growth, metastasis and therapy. Yet, little is known about spatial distribution patterns of these vessels. Most experimental or theoretical tumor models implicitly assume that blood vessels are equally abundant in different parts of the tumor, which has far-reaching implications for chemotherapy and tumor metabolism. In contrast, based on histological observations, we hypothesized that blood vessels follow specific spatial distribution patterns in colorectal cancer tissue. We developed and applied a novel computational approach to identify spatial patterns of angiogenesis in histological whole-slide images of human colorectal cancer.

A characteristic spatial pattern of blood vessels in colorectal cancer

In 33 of 34 (97%) colorectal cancer primary tumors blood vessels were significantly aggregated in a sharply limited belt-like zone at the interface of tumor tissue to the intestinal lumen. In contrast, in 11 of 11 (100%) colorectal cancer liver metastases, a similar hypervascularized zone could be found at the boundary to surrounding liver tissue. Also, in an independent validation cohort, we found this vascular belt zone: 22 of 23 (96%) samples of primary tumors and 15 of 16 (94%) samples of liver metastases exhibited the above-mentioned spatial distribution.

Summary and implications

We report consistent spatial patterns of tumor vascularization that may have far-reaching implications for models of drug distribution, tumor metabolism and tumor growth: luminal hypervascularization in colorectal cancer primary tumors is a previously overlooked feature of cancer tissue. In colorectal cancer liver metastases, we describe a corresponding pattern

and can be accessed via the following DOI: 10.5281/zenodo.260139.

Funding: JNK received financial support from Studienstiftung des Deutschen Volkes (no grant number, http://www.studienstiftung.de/en/). The other authors received no specific funding for this work. The funders had no role in study design, data collection and analysis, decision to publish, or preparation of the manuscript.

Competing interests: The authors have declared that no competing interests exist.

at the invasive margin. These findings add another puzzle piece to the complex concept of tumor heterogeneity.

Introduction

Spatial heterogeneity in solid tumors

Human solid tumors have a high degree of spatial heterogeneity, both on a genetic [1] and on a tissue level [2, 3]. This tumor heterogeneity is reflected by non-uniform distribution of microscopic structures of interest within a tumor; for example blood vessels [4], proliferating tumor cells [5] or lymphocytes in primary tumors [6, 7] and metastases [8, 9]. In general, heterogeneity can be appreciated in many scientific disciplines dealing with spatial data (e.g. earth sciences [10] or radiology [11, 12]). However, in cancer research and histological cancer diagnostics, this spatial heterogeneity is often neglected because of methodical limitations. For example, blood vessels and proliferating tumor cells are only counted in selected areas because quantification methods often rely on tedious manual procedures [13]. With the advent of digital pathology, microscopic objects in tumor tissue can be analyzed in a high-throughput manner in the entire spatial domain of a whole slide image (WSI) [2, 14]. Consequently, objects (such as blood vessels) and their features (e.g. size or shape) can be measured and subsequently visualized e.g. as a density map (heat map). Within these maps, one can easily appreciate the heterogeneity of the underlying data. Especially, "hotspot" regions of high density can be identified. However, for many applications in histopathology, there is no clear-cut definition of these hotspot areas. In this light, we have recently proposed a new method to analyze blood vessels that is based on spatial statistics and identifies all hotspot areas that are unlikely to occur by chance [4].

Blood vessel heterogeneity in tumors

As tumors grow, they induce the sprouting of blood vessels into the tumor tissue [15–17]. Spatial distribution of vessels (blood or lymphatic) has been investigated in some types of cancer [18–20]. Also, morphological parameters of intra-tumoral blood vessels have been linked to patient outcome in CRC and other types of cancer [21–24]. However, to our knowledge, there have been no systematic investigations on the spatial distribution of blood vessels in whole slide images (WSI) of CRC tissue except for our recent work on tumor angiogenic hotspots [4].

In this light, we investigated whether there are other characteristic distribution patterns of blood vessels in CRC tissue besides angiogenic hotspots.

Material and methods

Ethics statement

For this study, we retrieved randomly selected tumor tissue samples from N = 100 patients from our institution's pathology archive (S1 Fig). Prior to statistical analysis, we retrieved the TNM stage from the original pathology report (S1 Table). No other patient data was used within this study. For all analyses, the samples were fully anonymized. All experiments were approved by the institutional ethics board (medical ethics board II, University Medical Center Mannheim, Heidelberg University, Germany; approval 2015-868R-MA). The institutional ethics board waived the need for informed consent for this retrospective analysis of anonymized samples. All experiments were carried out in accordance with the Declaration of Helsinki.

Data availability

We release all raw data (images and measurement values) under a Creative Commons Attribution 4.0 License (http://creativecommons.org/licenses/by/4.0/). The data can be accessed via the following DOI: 10.17605/OSF.IO/D8AR6. This includes raw TIFF images of all N = 100 CRC samples and TIFF images of N = 5 normal colon mucosa.

Furthermore, we release N = 100 histological images that were used for validation of the segmentation procedure by human observers: DOI: 10.5281/zenodo.117997. This dataset includes the counts of three blinded observers and can be used by other groups to validate their own blood vessel segmentation algorithms.

Source codes used for this study are available under the MIT license (http://opensource.org/licenses/MIT) and can be accessed via the following DOI: 10.5281/zenodo.260139.

Sample collective

In this study, we analyzed two independent collectives of human tumor samples. We retrieved these tumor samples from our local pathology archive at University Medical Center Mannheim. In our archive search tool, we filtered samples by the keywords "colon carcinoma", "rectum carcinoma", "colectomy" and "hemicolectomy" and reviewed all search results manually. At this stage, we identified pathology reports that described a immunohistologically diagnosed microsatellite instability (MSI), a largely necrotic tumor or a tumor located in the appendix and omitted the corresponding samples. No further selection criteria were applied.

First cohort: We retrieved 65 unselected samples of CRC tissue from the local pathology archive of which four samples were excluded because of insufficient tissue quality, leaving N = 61 samples (see S1 Fig and S1 Table). From each patient, one tumor block was selected based on pre-existing hematoxylin & eosin (H&E) slides and was stained for CD34. One metastatic sample matched a primary tumor, i.e. was derived from the same patient (Smp006 and Smp029).

Validation cohort: The validation cohort was composed of N = 39 randomly selected CRC tumor samples, of which N = 23 were primary tumors and N = 16 were liver metastases. Two metastatic samples matched a primary (C2-Smp025 matched C2-Smp009 and C2-Smp028 matched C2-Smp014). To ensure homogeneity of this cohort, no neoadjuvantly pretreated tumors were included. Representative images from the validation cohort can be seen in S2 Fig.

Immunostaining and slide scanning

Immunohistochemistry (IHC) was performed using a routine immunoperoxidase technique [25, 26] with anti-CD34 antibody (Immunotech PN IM0786, 1:500), and with DAB (Diaminobenzidine) as chromogen. Subsequently, the slides were fully digitalized using an Aperio ScanScope (Aperio / Leica Biosystems, 20x objective magnification, approx. 0.5 μm per pixel) and saved as compressed Aperio SVS files, typically yielding 300 MB per slide. Whole slide images were reviewed and manually cropped, yielding TIFF images of 1–2 GB per sample (uncompressed).

Image analysis and object recognition

Intra-tumoral blood vessels were detected according to our previously published computational method [4], including color deconvolution [27], unsupervised thresholding [28] and morphological post-processing. To optimize computational performance, objects in the binary mask were processed using a dilation–erosion step without further complex morphological operations. On average, blood vessels had a major axis length of 12.7 μm ± 1.7 μm. The blood vessel detection method is explained in our previous publication [4].

The vessel segmentations method returned the centroid coordinates of recognized objects. These coordinates were passed on to our algorithm for automatic detection of angiogenic hotspots [4]. This hotspot detection is based on unsupervised kernel density estimation (KDE) [29]. Particularly, our previous publication includes a description of how the optimal threshold of significance for an angiogenic hotspot is calculated [4].

Analysis of spatial distribution patterns

To test whether objects in a histological whole slide image (WSI) were preferentially located close to the intestinal lumen or the invasion front, we devised and implemented a new spatial statistical approach. After object recognition, we manually delineated up to three ROIs in the WSI: "Tumor", "Adjacent Tissue" and "Intestinal Lumen". For each object in the "Tumor" ROI, we calculated the shortest distance to either of the two neighboring ROIs ("Adjacent Tissue" and "Intestinal Lumen"). For each image, this distribution of distances $D_{obs}(x)$ was compared to 100 control distributions $D^{i}_{ctrl}(x)$ (x = distance in mm, $i = \{i\epsilon\mathbb{N} \mid 1 \leq i \leq 100\}$). These control distributions followed complete spatial randomness (CSR). Blood vessel CSR patterns were created per a 2D Poisson process.

By comparing the observed pattern to the control pattern, we defined a spatial excess E of objects with respect to the distance from a region of interest (ROI) as follows:

$$E(x) = \frac{\sum_i D_{obs}(x) - D^{i}_{ctrl}(x)}{n}$$

(n being the number of independent repetitions of the experiment, default 100; x being the distance from "Lumen" or "Adjacent Tissue" ROIs in mm; i being the index variable for iterations from 1 to n). In this study, we use the term "excess" to denote an aggregation of objects of interest (e.g. blood vessels) in a specific region that statistically outnumbers the aggregation of these objects that can be expected under the null hypothesis of CSR.

Since the observed pattern and the control pattern contained an equal number of points, it follows that the distance distribution (visualized as a histogram) contained an equal number of object counts, so that

$$\int_0^\infty D_{obs}(x)dx = \int_0^\infty D_{ctrl}(x)dx$$

and by definition

$$\int_0^\infty E(x)dx = 0.$$

If the objects were aggregated close to the ROI, E(x) showed a positive peak close to x = 0 (seen in **Fig 1**B). If the objects were sparse close to the ROI, E(x) had negative values close to x = 0. We identified the first x-intersection x_0 of E(x) so that A as defined as

$$A = \int_0^{x_0} E(x)dx$$

was the absolute object excess close to the ROI (**Fig 1**B). In other words, E(x) was analyzed for the presence of a positive or negative object excess close to the ROI of interest. An object excess was considered statistically significant if the 95% confidence interval for the true mean did not contain zero. For a more detailed graphical explanation of the method, see S3 Fig.

Furthermore, we measured global descriptors of blood vessel distribution patterns as follows: in accordance with Vermeulen et al. [13], we counted blood vessels within angiogenic hotspots ("in-hotspot microvascular density (MVD)"). Additionally, we used the total number

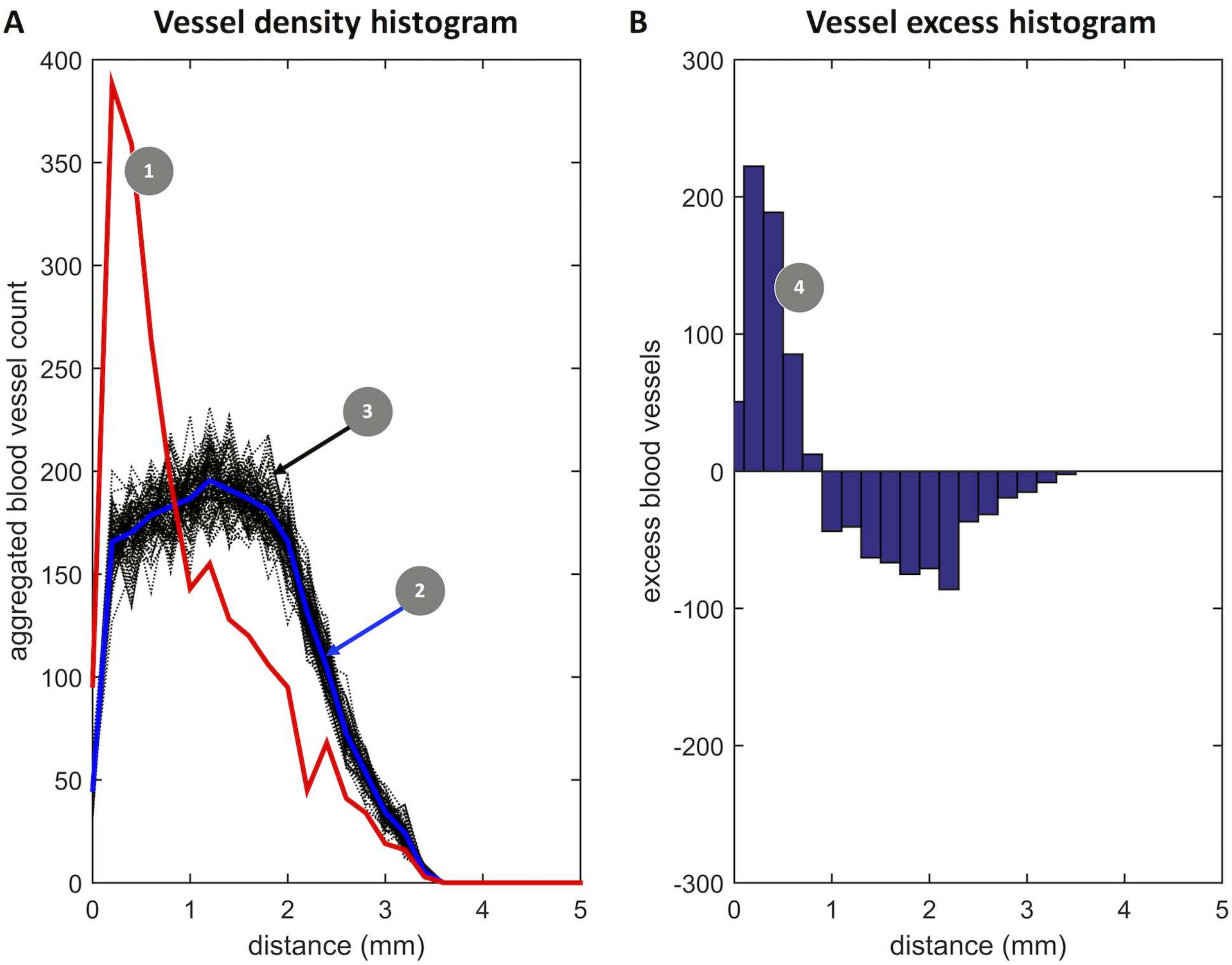

Fig 1. Graphical explanation of the "vessel excess". (A) The distance of each blood vessel to the intestinal lumen was measured. A histogram of these values is plotted in red (1). The red curve peaks at approx. 0.2 mm. This peak corresponds to an accumulation of blood vessels close to the intestinal lumen. We then assessed whether this peak is due to chance or due to a non-random effect. To this end, a random point pattern was simulated and the experiment was repeated with these random points. This was repeated 100 times. The results of these experiments are plotted in black (3), the mean distance histogram of the random points is shown in blue (2). The peak of the observed curve (1) is far outside the range of random fluctuations. This shows that the spatial accumulation of blood vessels close to the intestinal lumen is very likely not due to a random effect. (B) To quantify the blood vessel excess, the difference of (1) and (2) is plotted as a histogram. The area under the curve until the first x-intersection is regarded as the blood vessel excess close to the intestinal lumen. This Fig shows representative data for one sample (C2-Smp008).

doi:10.1371/journal.pone.0171378.g001

of blood vessels within the tumor ROI, subsequently denoted as "overall MVD". Also, the mean number of spatially separated hotspot areas per area was defined as "hotspot density".

Comparison to normal colon mucosa

For qualitative comparison of CRC vascularization patterns to normal colon mucosa, we randomly chose N = 5 anonymized patients from our sample collective and retrieved corresponding non-malignant colon tissue from our pathology archive.

Statistics and computational methods

All values are given as mean ± standard deviation except if otherwise noted. Uncertainty bars in waterfall plots denote the 95% confidence interval of the mean and uncertainty bars in other

plots denote standard deviation (see also the respective Fig legends). To test for significance, we used a two-tailed student's t-test with a level of significance of 0.05, except if otherwise noted. All computational methods in this study, e.g. image processing methods, spatial statistical calculations and data analysis were performed by self-developed MATLAB programs (MATLAB, Mathworks, Natick, MA, USA). For our experiments, we used a standard computer workstation (2.2 GHz Intel Core i7, 16 GB 495 DDR3 RAM). To analyze a whole slide image typically took between 20 and 40 minutes. No systematic effort was made to further optimize for computational performance. Further computational parameters are available in S1 List.

Results

Automatic blood vessel detection is equivalent to manual detection

In the present study, blood vessels were automatically detected in histopathological images. We validated our segmentation method by comparing automatic blood vessel counts in N = 100 histological image patches against three blinded human observers. The results of this experiment can be found in **Fig 2**. Here, it is evident that blood vessel counts by human observers show a certain inter-observer-variability (Pearson's correlation coefficient of observer (Obs.) 1 vs. Obs. 2: r = 0.98; Obs. 1 vs. Obs. 3: r = 0.93; Obs. 2 vs. Obs. 3: r = 0.95). Computer-based object recognition correlates well to the mean of human observers (r = 0.81). Although this correlation value is below the correlation between different human observers, a correlation of r = 0.81 generally indicates a strong positive relationship between two variables. We conclude that for the purposes of our study, the blood vessel segmentation method is sufficient.

Blood vessels in CRC primary tumors form an angiogenic zone close to the intestinal lumen

By visually inspecting continuous maps of intra-tumoral blood vessel density in CRC samples, we saw that angiogenic hotspots were accumulated in an area of the tumor facing the intestinal lumen in CRC primary tumors (**Fig 3**A and 3C). Conversely, in CRC metastases, we saw that angiogenic hotspots were predominantly located at the invasion front of the tumor (**Fig 3**B and 3D). Histologically, it was evident that these automatically detected hotspots align with tightly packed aggregates of dilated blood vessels (**Fig 4**C, S4 Fig) in primary tumors and aggregates of small vessels in metastases (**Fig 4**B). These aggregates were not present at the invasion front of primary tumors (**Fig 4**A). This observation led to our main hypothesis: "Blood vessels abundantly occur in a spatially distinct, non-random, vascular belt zone that is located close to the intestinal lumen in primary tumors and close to the invasion front in liver metastases". The next step was to test this hypothesis, objectively quantify these subjective observations and to assess the statistical significance of the observed pattern.

We developed and employed a statistical approach to find non-random spatial distribution patterns of intra-tumoral blood vessels in colorectal cancer samples. Initially, we automatically analyzed more than 300,000 vessels in N = 61 tumor tissue samples (50 primary tumors, 11 metastases; this is referred to as the first cohort). The intestinal lumen could be identified in N = 38 WSI of CRC primary tumor samples (of which N = 4 had undergone neoadjuvant treatment and N = 34 had not). In these N = 38 samples, we quantified the distribution of distances of blood vessels to the intestinal lumen. We found that in 33 out of 34 untreated CRC tumor samples (97%), blood vessels were significantly more numerous at the luminal side than would have been expected under a random spatial distribution (mean excess 457 ± 355 blood vessels, **Fig 5**A). The positive blood vessel excess was indeed restricted to a clearly defined zone of 1.27 ± 0.40 mm adjacent to the intestinal lumen. In pretreated CRC tumors, the

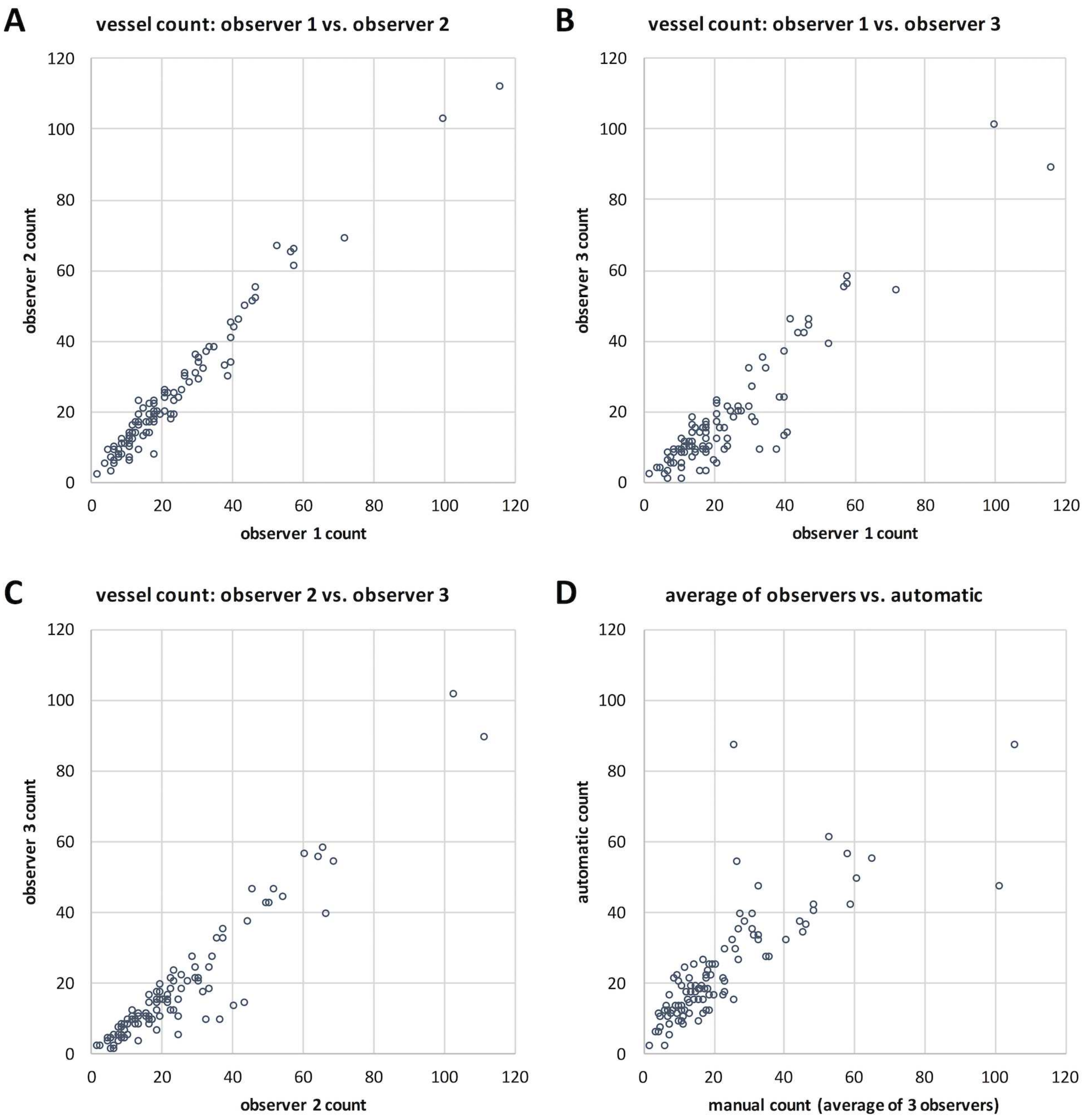

Fig 2. Validation of the segmentation procedure. Blood vessels in N = 100 image patches were counted by three blinded human observers. In each image patch, each observer manually determined the number of blood vessels. Then, for each image patch, these numbers were compared in a pair-wise manner between the three observers. The mean count calculated from the three observers was compared to the result of the automatic method. The experimental data are shown as scatter plots. (A-C) Inter-observer variability, (D) Correlation of the automatic count to the mean of human observers.

doi:10.1371/journal.pone.0171378.g002

pattern was different and a positive blood vessel excess at the luminal side was only observed in 2 of 4 samples and to a much lesser degree (mean excess 147 ± 265 blood vessels, width of the positive excess zone 1.02 ± 0.26 mm, Fig 5A)–although, because of the limited sample size of pretreated (neoadjuvant) CRC tumors, these numbers have to be viewed with caution.

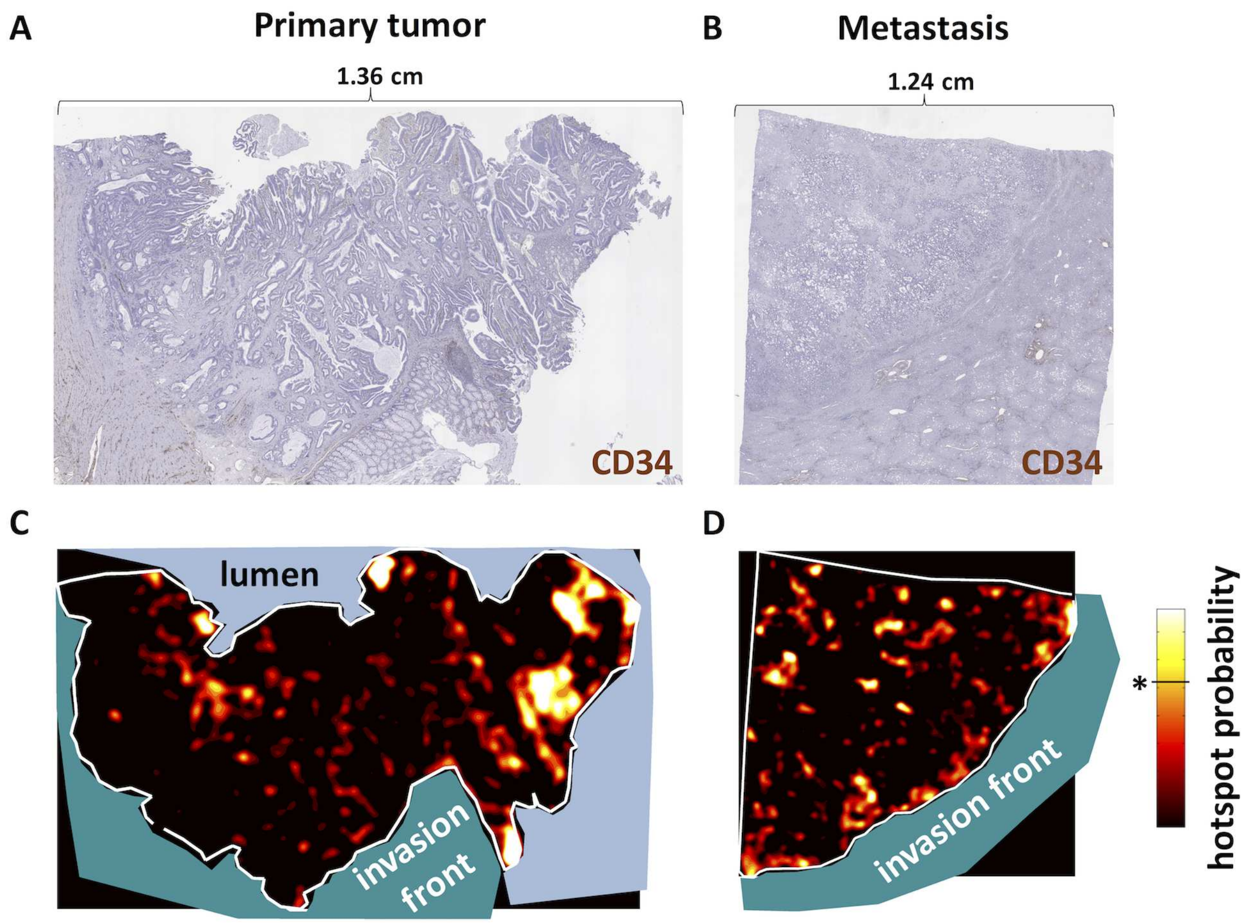

Fig 3. Characteristic vascular belt zones in CRC tissue. (A) Histological whole slide images of a representative primary tumor (B) and a liver metastasis stained for blood vessels (CD34). (C) The corresponding angiogenic hotspot probability map shows that angiogenic hotspots are preferably located close to the intestinal lumen in primary tumors. (D) Blood vessel distribution is more heterogeneous, but generally close to the invasion front in metastases (dark = low density, bright = high density, * in the color bar marks the level of significance).

doi:10.1371/journal.pone.0171378.g003

To exclude the possibility that the observed pattern is merely an artifact present in our sample collective, we assembled, stained and analyzed a second sample collective (referred to as validation cohort) of N = 39 human tumor samples (N = 16 metastases and N = 23 primary tumors). An overview of all samples is given in S1 Fig. A quantitative analysis of this cohort (**Fig 6**) completely reproduced the findings from the first cohort (**Fig 5**): 22 of 23 CRC primary tumors (96%) showed a significant positive blood vessel aggregation close to the intestinal lumen. Comparably to the samples in the first cohort, primary CRC tumor samples in the validation cohort exhibited a highly vascularized zone of mean width of 1.11 ± 0.37 mm. Mean blood vessel excess (as compared to a random spatial distribution) was 579 ± 450 blood vessels. These findings from the validation cohort are in qualitative and quantitative agreement with the first cohort.

Having analyzed the distribution of blood vessels relative to the intestinal lumen, we analyzed the distribution of blood vessels relative to the adjacent tissue (invasive margin). This is described in the following paragraph.

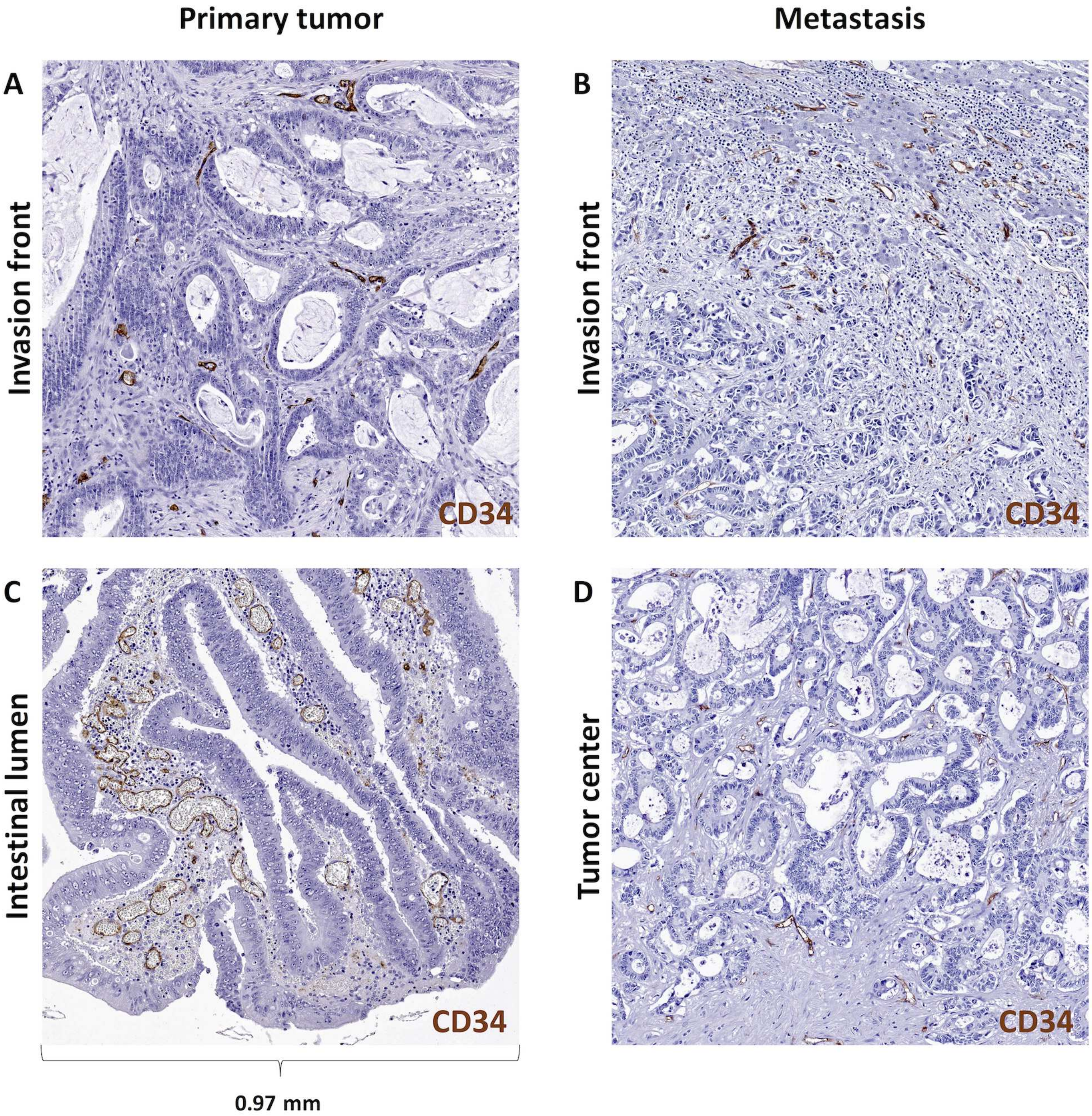

Fig 4. Histological aspect of the vascular belt zone in CRC tissue. CRC primary tumor sample stained for CD34 (brown), (A) primary tumor near the invasion front contains few blood vessels, (B) liver metastasis near the invasion front contains many small blood vessels, (C) primary tumor at the intestinal lumen contains many dilated blood vessels, (D) liver metastasis tumor center contains few blood vessels.

doi:10.1371/journal.pone.0171378.g004

Blood vessels in CRC liver metastases cluster in an angiogenic zone at the tumor invasion front

In the first cohort, the tumor invasion front was present in N = 51 of 61 histological whole slide images (among these: N = 36 untreated primary CRCs, N = 4 neoadjuvant CRCs, N = 11 CRC liver metastases, Fig 5B). We analyzed the distance of blood vessels to the adjacent tissue

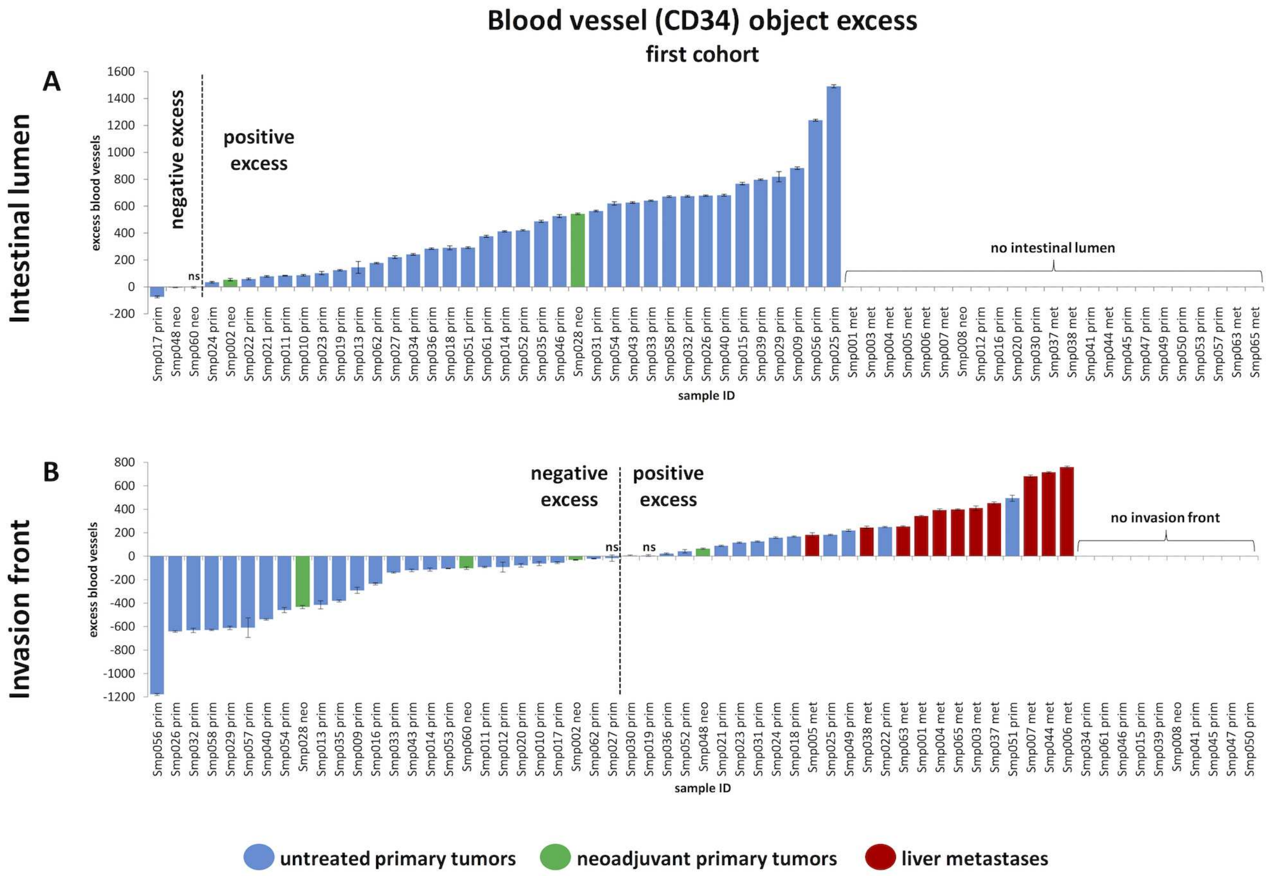

Fig 5. Magnitude of angiogenic zones for all analyzed samples in the first cohort, shown as waterfall plots. (A) Analysis of blood vessel aggregation in the tumor parts next to the intestinal lumen. Statistically significant positive blood vessel excess at the luminal side was detected in 33 of 34 untreated CRC primary tumors ("prim", blue) and 2 of 4 neoadjuvant CRC primary tumors ("neo", green). (B) Blood vessel excess at the invasion front in N = 36 untreated CRC primary tumors ("prim", blue), N = 4 neoadjuvant CRC primary tumors ("neo", green), and N = 11 CRC liver metastases ("met", red). (A-B) Error bars indicate the 95% confidence interval that was calculated by a Monte Carlo method. All measurements are statistically significant except if labeled "ns" (for "not significant").

doi:10.1371/journal.pone.0171378.g005

and found a marked positive blood vessel excess at the invasion front in all 11 of 11 metastases (mean excess 440 ± 198 blood vessels, Fig 5B). The mean thickness of this blood vessel excess zone was 1.36 ± 0.37 mm. In untreated primary CRCs, we observed a significant positive blood vessel excess at the invasion front in only 12 of 36 samples (33%). In these 12 samples, the mean excess in the positive excess zone was 156 ± 131 vessels and the mean thickness of the positive excess zone was 0.78 ± 0.37 mm. In short, while a vascular belt zone close to the adjacent tissue was observed in all metastases, only 33% of untreated primary CRCs exhibited a positive blood vessel excess zone and this zone was much thinner and less pronounced than in CRC metastases (Fig 5B and Fig 6B).

Like before, we validated these findings in our validation cohort. The tumor invasion front was present in all but one (38 of 39) sample in this cohort (N = 23 primary tumors and N = 16 metastases). As reflected in the primary cohort only a few (3 of 22, 14%) primary tumors exhibited a blood vessel excess close to the invasion front. Conversely, 15 of 16 (94%) of all liver metastases in the validation cohort showed this specific pattern. Thus, the findings in the validation cohort completely reproduced the findings from the first cohort.

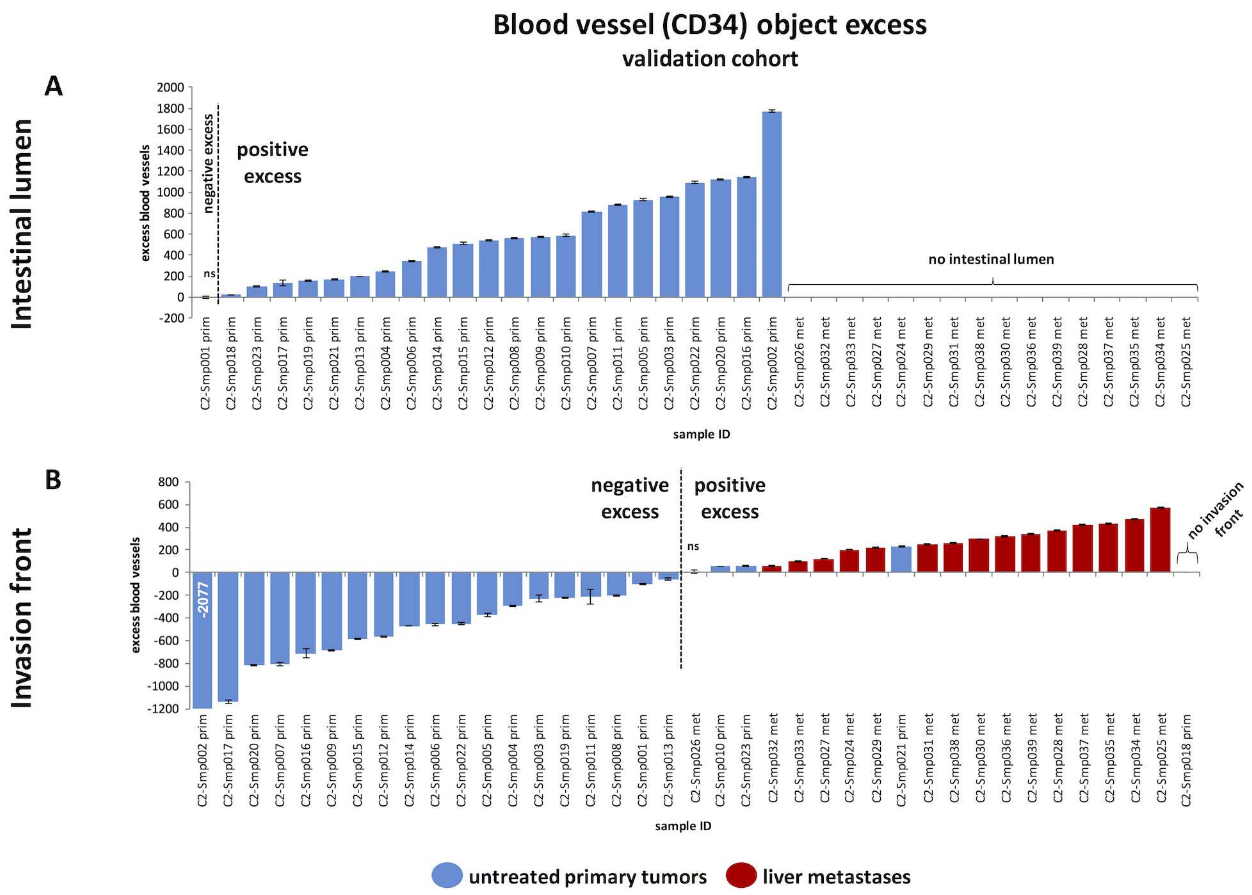

Fig 6. Magnitude of angiogenic zones for all analyzed samples in the validation cohort, shown as waterfall plots. This Fig shows the result of the analysis of the independent validation cohort of N = 39 samples and is organized identically to Fig 5. (A) Analysis of blood vessel aggregation in the tumor parts next to the intestinal lumen. (B) Blood vessel excess at the invasion front. The first sample was cropped at -1200, but the true value of -2077 is overlaid on the bar. (A-B) Error bars indicate the 95% confidence interval that was calculated by a Monte Carlo method. All measurements are statistically significant except if labeled "ns" (for "not significant").

doi:10.1371/journal.pone.0171378.g006

In summary (as sketched in Fig 7), liver metastases and primary tumors exhibited quite different spatial distribution patterns of blood vessels close to the invasive margin. This difference in "adjacent tissue blood vessel excess" was highly unlikely a product of chance ($p < 0.001$).

Different angiogenic activity in metastases than in primary tumors

In the present study, we could replicate our previous finding that angiogenic hotspots are spatially clearly defined, contiguous areas in a tumor image [4]. We quantified the size and number of these hotspot areas in all samples and found pronounced differences between primary tumors and metastases (given for the combined data set consisting of both cohorts, excluding neoadjuvantly pretreated tumors): In liver metastases, hotspot density was much higher while overall microvascular density (MVD) was markedly lower than in primary tumors: Hotspot density was 0.37 ± 0.13 hotspots/mm^2 in untreated primary tumors (N = 68) and 0.44 ± 0.17 in metastases (N = 27), with p = 0.026. MVD was 84 ± 22 vessels/mm^2 in primaries (N = 68) and 74 ± 19 vessels/mm^2 in metastases (N = 27), with p = 0.046.

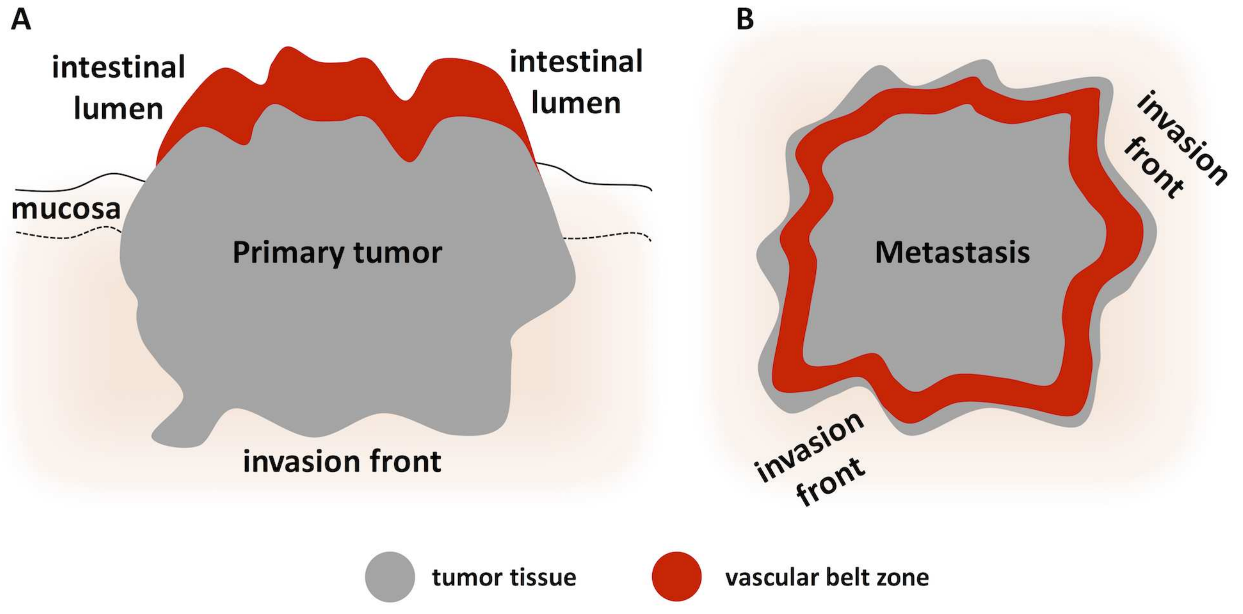

Fig 7. Graphical summary of the model. (A-B) Based on experimental data, we propose a new model for angiogenesis that is schematically shown in A for primary tumors and B for metastasis. The model states that blood vessels are preferentially located close to the intestinal lumen in primary tumors and close to the invasion front in liver metastases.

doi:10.1371/journal.pone.0171378.g007

Comparison of total microvascular density, in-hotspot microvascular density and number of hotspots

In-hotspot MVD is a widely-used parameter to assess tumor vascularization [13], but it is unclear how it correlates to overall MVD. We analyzed both parameters in N = 100 samples (first cohort and validation cohort) and found that in metastases and primary tumors, overall MVD and in-hotspot MVD are well correlated (Pearson's correlation coefficient r = 0.67, S5 Fig).

Discussion

Characteristic angiogenic patterns in CRC tissue

In the present study, we investigated spatial patterns of blood vessel distribution in human CRC samples for the first time and found characteristic patterns: (1) intra-tumoral blood vessels were aggregated in a clearly defined zone close to the intestinal lumen in primary tumors. (2) Conversely, in liver metastases, blood vessels aggregated at the tumor invasion front. We describe this aggregation of blood vessels as an "excess", because it is statistically significantly higher than expected under the null hypothesis (see Material and Methods section). However, this term does not imply that these blood vessels are "unnecessary"–rather, the biological significance of this pattern must be analyzed in further studies. Some of the possible hypotheses that arise from this pattern are discussed below: For example, the unexpected juxtaposition of the angiogenic belt to the gut lumen could not be explained by ulceration or chronic inflammation in histopathological re-evaluation of the slides.

Possible clinical implications and perspectives

These findings led us to a new model of tumor vascularization (Fig 7A and 7B): a highly vascularized zone of approx. 1.5 mm width is present close to the intestinal lumen in CRC primary

tumors. Also, a highly vascularized zone of approx. 1 mm width is present close to the invasion front in CRC liver metastases. This model has several potential implications:

1. Tumor-host-interaction at the intestinal lumen: Several studies have proposed mechanisms of tumor-host interaction of CRC at the basolateral [30–32] and on the luminal side [33, 34]. Our findings show that CRC tissue facing the intestinal lumen is usually hypervascularized. This raises new questions about interactions at this side of the tumor, e.g. about cancer-microbiome-interactions. It is conceivable that luminal hypervascularization influences diffusion of bacteria-derived or nutrition-derived molecules into the tumor tissue, but vice versa these factors could also contribute to the formation of this new type of spatial vascular heterogeneity in CRC. Based on our findings, it would be highly interesting to investigate molecular interactions between the microbiome, nutrition and the luminal tumor vasculature. Furthermore, it would be interesting to analyze in future studies tumors of hollow organs that face a lumen with a sterile fluid like transitional cell carcinoma of the bladder.

2. Hypoxia: Tumor vascularization determines tumor hypoxia, which triggers a plethora of signaling pathways [35–37]. Previously, Righi et al. have shown that tumors with a relative lack of blood vessels at the invasion front are hypoxic in this area and that this is associated with increased tumor cell budding [38]. Our findings significantly extend this study since we demonstrate that in primary CRCs, vascular patterns at the invasion front markedly differ between two groups of tumors: One group has a relative lack of blood vessels at the tumor invasion front (i.e. a "negative excess") and another group of CRC primary tumors that shows a metastasis-like vascularization pattern with a positive blood vessel excess at the invasion front (**Fig 5**B, **Fig 6**B).

3. Metastasis: Liver metastasis in CRC is caused by tumor cells that successfully invade the blood stream and subsequently the liver parenchyma [39]. Our experimental results suggest that CRC tumor cells growing close to the intestinal lumen may have a better access to blood vessels than tumor cells in other regions. Further studies should explore how this property of tumors influences the propensity of tumor cells to metastasize to the liver.

4. Distribution of chemotherapy drugs in tumor tissue: Vascular patterns determine how chemotherapeutic drugs are distributed in tumor tissue [40]. Consequently, according to our data it is likely that such drugs reach the luminal side of CRC tumors much easier than the basolateral side. However, the heterogeneous spatial distribution of blood vessels is generally not considered when thinking about the effects of chemotherapy in tumor tissue [41]. Considering quantitative spatial vascularization patterns could help to optimize tumor therapy (e.g. timing of surgery, since the tumor parts of the deep invasion front may be less sensitive to chemotherapy).

5. Anti-angiogenic therapy: Anti-angiogenic therapy has been proved to have beneficial effects in some CRC patients, but these effects are limited [42–44]. How spatial patterns of blood vessel distribution might influence the effectivity of this therapy in CRC is currently unknown.

6. Early symptoms of CRC: From a clinical perspective, the presence of an angiogenic belt zone at the intestinal lumen is a conceivable explanation for the common clinical observation that gastrointestinal bleeding is a frequent symptom of colon cancer and that in consequence, tests for occult fecal blood are useful and sensitive.

7. Architecture of CRC in comparison to normal colon mucosa: Normal colon mucosa is a highly structured, well-vascularized tissue. In **Fig 8**, the normal blood vessel pattern in

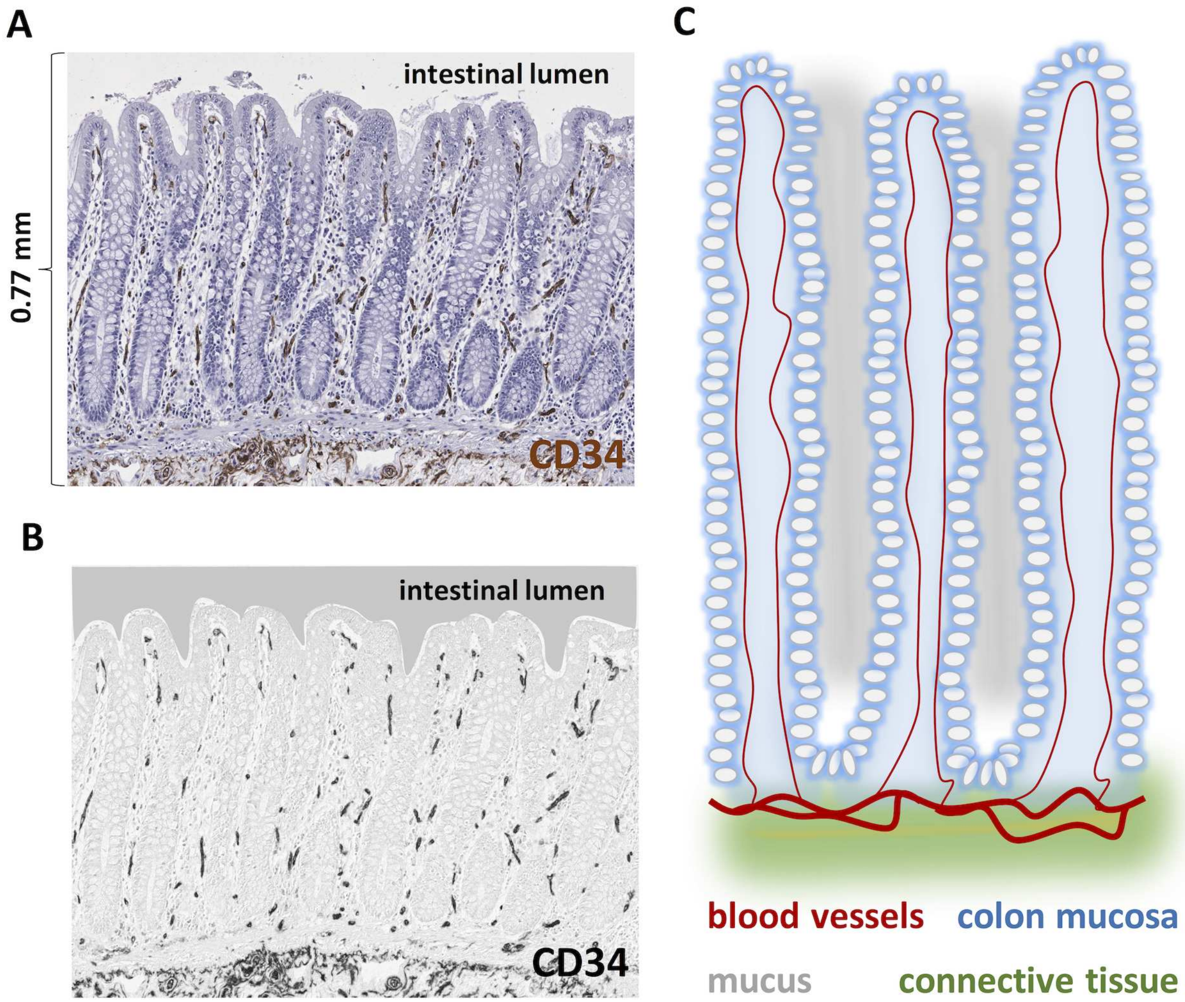

Fig 8. Physiological blood vessel distribution in colon mucosa. Blood vessel architecture in normal colon mucosa where blood vessels run along the crypt wall at the barrier to the intestinal lumen. (A) Original CD34-stained image. This image is representative of five independent whole slide images. (B) For better clarity, the original image was processed by color deconvolution and only the CD34-staining is shown. (C) Schematic illustration of the physiological blood vessel architecture in colon mucosa. A blood vessel runs closely to the surface between two adjacent crypts.

doi:10.1371/journal.pone.0171378.g008

colon mucosa is shown (described before by Kachlik et al. [45], reproduced in N = 5 WSI in the present study). Our finding that blood vessels in CRC are preferentially located close to the intestinal lumen qualitatively corresponds to the known blood vessel distribution in normal colon mucosa. (Note that it is not possible to quantitatively analyze blood vessel hotspots in normal colon mucosa in the same way as in CRC because normal colon mucosa is much thinner [approx. 700μm] than CRC tissue [several mm to cm] and because its vascular architecture is governed by the structured architecture with crypts).

It is conceivable that CRC tissue inherits the blood vessel architecture from non-neoplastic normal colon mucosa. In further studies, it could be investigated whether this phenotypical resemblance of vascularization pattern also corresponds to functional similarities in pro-angiogenic signaling.

Limitations, reproducibility and outlook

We describe a novel angiogenic belt zone in CRC with potential clinical implications. Still, the presented results reflect a morphological finding that raises the need for functional investigations. There are three different compartments in a CRC primary tumor that can be clearly distinguished according to their vascularization pattern: a luminal angiogenic belt zone, a tumor bulk and, in a minority of samples, a thin angiogenic belt at the invasion front. Comparative analysis of these three compartments by 3D-histology, mass-spectrometry and (epi-)genetic analysis following micro-dissection will further elucidate the biological role of different spatial patterns in CRC. Furthermore, future studies could evaluate the described spatial patterns in the light of prognostic biomarkers. This could be addressed by combining survival data with morphological measurements.

Our claim that the spatial patterns are universal features of CRC tissue are based on two independent sample cohorts of a total N = 100 tumors. Still, our findings and our thereon based hypothesis should be validated by other researchers: During our experiments, we noted that the vascular belt zone can be identified by thorough visual examination of CD34-immunostained histological slides (see, for example, **Fig 4** and S2 Fig)—and in some cases even in H&E stained samples (not shown). Thus, we expect that visual examination (without full quantitative analysis) is sufficient to qualitatively reproduce our findings (still, of course, we provide all our raw data and source code for others to use, see below).

Finally, it is worth mentioning that the current study focuses on the tumor proper. Analogous spatial statistical studies of the peritumoral microenvironment are obviously mandatory in CRC as well.

Supporting information

S1 Table. CRC patient characteristics of both cohorts. Sample IDs starting with "C2" are from the second cohort (validation cohort). Four samples from the primary cohort were excluded after staining (these are not shown): Smp042 was an appendix carcinoma, Smp055 was a lymph node metastasis, Smp059 was a duplicate of Smp058 and Smp064 was insufficiently stained and were removed after assignment of sample numbers. T = local tumor stage, N = lymph node stage, M = distant metastasis stage at time of surgery. In total, N = 100 samples were analyzed.
(DOCX)

S1 Fig. Overview of the sample collective. Here, the two cohorts are schematically shown.
(TIF)

S2 Fig. Representative images from the validation cohort. Image patches of 2.25 mm^2 close to the intestinal lumen and close to the invasion front are shown, with A matching D, B matching E and C matching F. All three samples are CRC primary tumors. Even in this low magnification, blood vessels (CD34-positive) are much more abundant in the image patches close to the intestinal lumen. As seen in D (and, to a lesser extent, in E and F), these blood vessels are usually large and dilated.
(TIF)

S3 Fig. Flowchart of the computational methods for spatial statistics. (A) Comparison of observed spatial distribution patterns to random patterns. (1) Detail from the original WSI (Smp040), scale bar 300 μm, CD34-immunostaining labels blood vessel endothelium. The area is densely packed with blood vessels. (2) In the WSI, three regions are manually defined: intestinal lumen (green), tumor (black), adjacent tissue (orange). Blood vessels in the WSI are

automatically detected and plotted as white points. (3) To generate an internal control, detected blood vessels in a ROI are randomly redistributed N = 100 times using a Monte Carlo procedure so that overall MVD remains constant. (4) For each blood vessel, the distance to the intestinal lumen is calculated. This is repeated for each random pattern. The actual distribution of distances is compared to the distribution of random distances, plotted as a histogram. (B) Identification of excess zones: (5) The distance distribution of the random patterns is subtracted from the distance distribution of the observed patterns. The resulting difference is plotted as an excess histogram E(x). The first peak in E(x) reflects an excess of blood vessels at the luminal side. (6) The width of this peak describes the width of the excess zone while the total peak area describes the excess amount. (7) The excess amount is represented as one bar in a waterfall plot.
(TIF)

S4 Fig. Blood vessel morphology. (A) Angiogenic hotspot probability map in a representative sample (Smp040), (*) refers to the level of significance. **(B)** Each blood vessel within the tumor is represented by a filled circle. The bright gray region shows the adjacent tissue and the dark gray region shows the intestinal lumen. As shown above, blood vessels tend to cluster at the intestinal lumen. Additionally, the solidity (defined as area divided by convex area) is shown for each blood vessel (blue = less solid, yellow = solid). Most non-solid vessels are located at the luminal side. This corresponds to the morphological observation that intratumoral blood vessels at the intestinal lumen tend to be more dilated than blood vessels in other tumor regions.
(TIF)

S5 Fig. In-hotspot microvascular density (MVD) is a suitable surrogate parameter of overall MVD. Scatter plot of overall MVD vs. in-hotspot of all samples (N = 100, both cohorts). Pearson's correlation coefficient is r = 0.67.
(TIF)

S1 List. List of computational parameters.
(DOCX)

Acknowledgments

We are grateful to Prof. Schultz of the department of Neuroanatomy at Medical Faculty Mannheim for the cooperation. Also, we want to thank Ms. Katrin Wolk, Ms. Barbara Froehlich and Mr. Yannick Bukschat for expert technical assistance.

Author Contributions

Conceptualization: JNK FGZ CAW.

Data curation: JNK FGZ CAW AM TG.

Formal analysis: JNK FGZ LRS SMM CAW.

Funding acquisition: JNK CAW TG AM FGZ LRS HPS.

Investigation: JNK CAW.

Methodology: JNK CAW.

Project administration: JNK FGZ CAW.

Resources: LRS HPS AM TG.

Software: JNK FGZ SMM CAW.

Supervision: LRS AM TG HPS.

Validation: JNK SMM HPS AM TG CAW.

Visualization: JNK CAW.

Writing – original draft: JNK CAW AM TG FGZ.

Writing – review & editing: JNK FGZ LRS SMM AM TG CAW.

References

1. Gerlinger M, Rowan AJ, Horswell S, Larkin J, Endesfelder D, Gronroos E, et al. Intratumor heterogeneity and branched evolution revealed by multiregion sequencing. N Engl J Med. 2012; 366(10):883–92. doi: 10.1056/NEJMoa1113205 PMID: 22397650
2. Heindl A, Nawaz S, Yuan Y. Mapping spatial heterogeneity in the tumor microenvironment: a new era for digital pathology. Lab Invest. 2015; 95(4):377–84. doi: 10.1038/labinvest.2014.155 PMID: 25599534
3. Stahl PR, Schnellert J, Koop C, Simon R, Marx A, Izbicki JR, et al. Determination of Tumor Heterogeneity in Colorectal Cancers Using Heterogeneity Tissue Microarrays. Pathol Oncol Res. 2015.
4. Kather JN, Marx A, Reyes-Aldasoro CC, Schad LR, Zöllner FG, Weis C-A. Continuous representation of tumor microvessel density and detection of angiogenic hotspots in histological whole-slide images. Oncotarget. 2015; 6(22):19163–76. doi: 10.18632/oncotarget.4383 PMID: 26061817
5. Waclaw B, Bozic I, Pittman ME, Hruban RH, Vogelstein B, Nowak MA. A spatial model predicts that dispersal and cell turnover limit intratumour heterogeneity. Nature. 2015; 525(7568):261–4. doi: 10.1038/nature14971 PMID: 26308893
6. Gooden MJ, de Bock GH, Leffers N, Daemen T, Nijman HW. The prognostic influence of tumour-infiltrating lymphocytes in cancer: a systematic review with meta-analysis. Br J Cancer. 2011; 105(1):93–103. doi: 10.1038/bjc.2011.189 PMID: 21629244
7. Yuan Y, Failmezger H, Rueda OM, Ali HR, Graf S, Chin SF, et al. Quantitative image analysis of cellular heterogeneity in breast tumors complements genomic profiling. Sci Transl Med. 2012; 4(157):157ra43.
8. Halama N, Michel S, Kloor M, Zoernig I, Benner A, Spille A, et al. Localization and density of immune cells in the invasive margin of human colorectal cancer liver metastases are prognostic for response to chemotherapy. Cancer Res. 2011; 71(17):5670–7. doi: 10.1158/0008-5472.CAN-11-0268 PMID: 21846824
9. Halama N, Spille A, Lerchl T, Brand K, Herpel E, Welte S, et al. Hepatic metastases of colorectal cancer are rather homogeneous but differ from primary lesions in terms of immune cell infiltration. Oncoimmunology. 2013; 2(4):e24116. doi: 10.4161/onci.24116 PMID: 23734335
10. Wainwright J, Mulligan M. Environmental modelling: finding simplicity in complexity: John Wiley & Sons; 2005.
11. Schad LR, Bluml S, Zuna I. MR tissue characterization of intracranial tumors by means of texture analysis. Magn Reson Imaging. 1993; 11(6):889–96. PMID: 8371644
12. Domsch S, Mie MB, Wenz F, Schad LR. Non-invasive multiparametric qBOLD approach for robust mapping of the oxygen extraction fraction. Z Med Phys. 2014; 24(3):231–42. doi: 10.1016/j.zemedi.2014.03.009 PMID: 24743060
13. Vermeulen PB, Gasparini G, Fox SB, Colpaert C, Marson LP, Gion M, et al. Second international consensus on the methodology and criteria of evaluation of angiogenesis quantification in solid human tumours. Eur J Cancer. 2002; 38(12):1564–79. PMID: 12142044
14. Ghaznavi F, Evans A, Madabhushi A, Feldman M. Digital imaging in pathology: whole-slide imaging and beyond. Annu Rev Pathol. 2013; 8:331–59. doi: 10.1146/annurev-pathol-011811-120902 PMID: 23157334
15. Carmeliet P, Jain RK. Angiogenesis in cancer and other diseases. Nature. 2000; 407(6801):249–57. doi: 10.1038/35025220 PMID: 11001068
16. Ferrara N, Kerbel RS. Angiogenesis as a therapeutic target. Nature. 2005; 438(7070):967–74. doi: 10.1038/nature04483 PMID: 16355214
17. Kerbel RS. Tumor angiogenesis. N Engl J Med. 2008; 358(19):2039–49. doi: 10.1056/NEJMra0706596 PMID: 18463380

18. Balsat C, Blacher S, Signolle N, Beliard A, Munaut C, Goffin F, et al. Whole slide quantification of stromal lymphatic vessel distribution and peritumoral lymphatic vessel density in early invasive cervical cancer: a method description. ISRN Obstet Gynecol. 2011; 2011(354861).
19. Ekdawi SN, Stewart JMP, Dunne M, Stapleton S, Mitsakakis N, Dou YN, et al. Spatial and temporal mapping of heterogeneity in liposome uptake and microvascular distribution in an orthotopic tumor xenograft model. J Control Release. 2015; 207:101–11. doi: 10.1016/j.jconrel.2015.04.006 PMID: 25862513
20. Muralidharan V, Nguyen L, Banting J, Christophi C. The prognostic significance of lymphatics in colorectal liver metastases. HPB Surg. 2014; 2014:954604. doi: 10.1155/2014/954604 PMID: 24963215
21. Caie PD, Turnbull AK, Farrington SM, Oniscu A, Harrison DJ. Quantification of tumour budding, lymphatic vessel density and invasion through image analysis in colorectal cancer. J Transl Med. 2014; 12:156. doi: 10.1186/1479-5876-12-156 PMID: 24885583
22. Des Guetz G, Uzzan B, Nicolas P, Cucherat M, Morere JF, Benamouzig R, et al. Microvessel density and VEGF expression are prognostic factors in colorectal cancer. Meta-analysis of the literature. Br J Cancer. 2006; 94(12):1823–32. doi: 10.1038/sj.bjc.6603176 PMID: 16773076
23. Jayasinghe C, Simiantonaki N, Kirkpatrick CJ. Histopathological features predict metastatic potential in locally advanced colon carcinomas. BMC Cancer. 2015; 15(1):1–14.
24. Piña Y, Cebulla CM, Murray TG, Alegret A, Dubovy SR, Boutrid H, et al. Blood vessel maturation in human uveal Melanoma: Spatial distribution of neovessels and mature vasculature. Ophthalmic Res. 2009; 41(3):160–9. doi: 10.1159/000209670 PMID: 19321938
25. Bender JL, Yue RYK, To MJ, Deacken L, Jadad AR. A lot of action, but not in the right direction: Systematic review and content analysis of smartphone applications for the prevention, detection, and management of cancer. Journal of Medical Internet Research. 2013; 15.
26. Welsch U, Mulisch M. Romeis Mikroskopische Technik: Spektrum Akademischer Verlag; 2010.
27. Ruifrok AC, Johnston DA. Quantification of histochemical staining by color deconvolution. Anal Quant Cytol Histol. 2001; 23(4):291–9. PMID: 11531144
28. Yen JC, Chang FJ, Chang S. A new criterion for automatic multilevel thresholding. IEEE Trans Image Process. 1995; 4(3):370–8. doi: 10.1109/83.366472 PMID: 18289986
29. Botev ZI, Grotowski JF, Kroese DP. Kernel density estimation via diffusion. Ann Stat. 2010; 38 (5):2916–57.
30. Egeblad M, Nakasone ES, Werb Z. Tumors as organs: complex tissues that interface with the entire organism. Dev Cell. 2010; 18(6):884–901. Epub 2010/07/16. doi: 10.1016/j.devcel.2010.05.012 PMID: 20627072
31. Zlobec I, Lugli A. Invasive front of colorectal cancer: Dynamic interface of pro-/anti-tumor factors. World J Gastroenterol. 2009; 15(47):5898–906. doi: 10.3748/wjg.15.5898 PMID: 20014453
32. Peddareddigari VG, Wang D, Dubois RN. The tumor microenvironment in colorectal carcinogenesis. Cancer Microenviron. 2010; 3(1):149–66. doi: 10.1007/s12307-010-0038-3 PMID: 21209781
33. Tjalsma H, Boleij A, Marchesi JR, Dutilh BE. A bacterial driver-passenger model for colorectal cancer: beyond the usual suspects. Nat Rev Microbiol. 2012; 10(8):575–82. doi: 10.1038/nrmicro2819 PMID: 22728587
34. Burns MB, Lynch J, Starr TK, Knights D, Blekhman R. Virulence genes are a signature of the microbiome in the colorectal tumor microenvironment. Genome Med. 2015; 7(1):55. doi: 10.1186/s13073-015-0177-8 PMID: 26170900
35. Raman V, Artemov D, Pathak AP, Winnard PT, McNutt S, Yudina A, et al. Characterizing Vascular Parameters in Hypoxic Regions: A Combined Magnetic Resonance and Optical Imaging Study of a Human Prostate Cancer Model. Cancer Res. 2006; 66(20):9929–36. doi: 10.1158/0008-5472.CAN-06-0886 PMID: 17047055
36. Østergaard L, Tietze A, Nielsen T, Drasbek KR, Mouridsen K, Jespersen SN, et al. The Relationship between tumor blood flow, angiogenesis, tumor hypoxia, and aerobic glycolysis. Cancer Res. 2013; 73 (18):5618–24. doi: 10.1158/0008-5472.CAN-13-0964 PMID: 23764543
37. Rijken PFJW, Bernsen HJJA, Peters JPW, Hodgkiss RJ, Raleigh JA, van der Kogel AJ. Spatial relationship between hypoxia and the (perfused) vascular network in a human glioma xenograft: a quantitative multi-parameter analysis. Int J Radiat Oncol Biol Phys. 2000; 48(2):571–82. PMID: 10974478
38. Righi A, Sarotto I, Casorzo L, Cavalchini S, Frangipane E, Risio M. Tumour budding is associated with hypoxia at the advancing front of colorectal cancer. Histopathology. 2015; 66(7):982. doi: 10.1111/his.12602 PMID: 25381897
39. Langley RR, Fidler IJ. The seed and soil hypothesis revisited-The role of tumor-stroma interactions in metastasis to different organs. Int J Cancer. 2011; 128(11):2527–35. doi: 10.1002/ijc.26031 PMID: 21365651

40. Primeau AJ, Rendon A, Hedley D, Lilge L, Tannock IF. The distribution of the anticancer drug Doxorubicin in relation to blood vessels in solid tumors. Clin Cancer Res. 2005; 11(24 Pt 1):8782–8. Epub 2005/12/20.
41. Brannon-Peppas L, Blanchette JO. Nanoparticle and targeted systems for cancer therapy. Adv Drug Deliv Rev. 2012; 64:206–12.
42. Nguyen L, Fifis T, Malcontenti-Wilson C, Chan LS, Costa PNL, Nikfarjam M, et al. Spatial morphological and molecular differences within solid tumors may contribute to the failure of vascular disruptive agent treatments. BMC Cancer. 2012; 12(1):522-.
43. Tozer GM. Measuring tumour vascular response to antivascular and antiangiogenic drugs. Br J Radiol. 2003; 76(suppl_1):S23–S5.
44. Bergers G, Hanahan D. Modes of resistance to anti-angiogenic therapy. Nature Reviews Cancer. 2008; 8(8):592–603. doi: 10.1038/nrc2442 PMID: 18650835
45. Kachlik D, Baca V, Stingl J. The spatial arrangement of the human large intestinal wall blood circulation. J Anat. 2010; 216(3):335–43. doi: 10.1111/j.1469-7580.2009.01199.x PMID: 20447248
